# Research highlights in neurorehabilitation

**DOI:** 10.1186/1743-0003-11-21

**Published:** 2014-03-04

**Authors:** Marta Pajaro-Blázquez, Jose Luis Pons

**Affiliations:** 1 Spanish National Research Council (CSIC), Bioengineering Group, Ctra. Campo Real km 0.200, La Poveda, 28500 Madrid, Arganda del Rey, Spain

## Abstract

Recent studies of the mechanisms underlying plasticity and recovery following neurological injuries have originated innovative lines of research in neurorehabilitation. Additionally, the development of new technologies to facilitate the performance of evaluation and intervention procedures has stimulated research on novel rehabilitation paradigms and more effective rehabilitation strategies. However, translation of novel interventions into clinical practice remains a challenge. Further investigation to evaluate the effectiveness of novel rehabilitation approaches is needed. In this thematic series, six manuscripts summarize the results of current research with focus on evaluation and treatment strategies of relevance in neurorehabilitation.

## Editorial

Many relevant areas in the field of neurorehabilitation (NR) have witnessed significant developments over the last two decades. Nevertheless, there are still major challenges that neuroscientists need to address to achieve clinically-relevant results in this field. The manuscripts included in this thematic series deal with two main problems in NR:

1) The development of accurate assessment tools for the evaluation of neurological deficits, such as spasticity, sensorimotor impairments, weakness and functional limitations.

2) Improvement in therapies aimed at both minimizing chronic deficits and maximizing function after central nervous system (CNS) injury.

On one hand, relatively simple techniques that can be easily utilized in the clinic to assess the severity of injuries of the CNS that affect descending motor commands and thus movement control are still lacking. These tools should allow clinicians to accurately diagnose and characterize different deficits and detect changes over time. In addition, the evaluation procedures should be easy to implement and hence applicable in a clinical setting. The outcomes of such evaluation procedures would be used to guide therapies, predict prognosis and monitor changes [1]. On the other hand, robotic and sensor-based systems enabling novel therapies have emerged and quickly gained the attention of researchers and clinicians. These technologies provide the means to deliver intensive therapies and to lessen the physical effort required by clinical personnel during manual therapy. However, there is no clear evidence that better outcomes can be achieved using technology-assisted intervention modalities (such as robotic therapy) than with conventional therapies. This might be partially due to the fact that active participation of patients throughout the rehabilitative process is a prerequisite to facilitate recovery. The first robotic devices that were made available for clinical studies and subsequently for clinical interventions were predominantly systems that completely guided the subjects’ movements while the user remained passive during the therapy. Robotic systems recently developed for application in rehabilitation provide different control strategies that are designed to promote subjects’ engagement and participation during therapy. To achieve this goal, in addition to carrying out clinical research studies with focus on the assessment of different robotic and non-robotic technologies, researchers need to gain a better understanding of the processes underlying motor recovery and motor adaptation, and leverage scientific discoveries in these areas to develop new control strategies for both robotic and sensor-based devices [2].

The 2012 International Conference in Neurorehabilitation (ICNR2012) was recently held in Toledo (November 14–16, 2012) and was partially founded by the HYPER project (Spanish CONSOLIDER-INGENIO 2010 programme, CSD2009-00067). ICNR2012 aimed to bring together scientists from multiple disciplines with a common interest in Neural Engineering and Rehabilitation. The meeting provided a suitable scientific environment to discuss the latest advances in the field. Assessment and treatment strategies in NR were the focus of many contributions many presentations at the conference [3]. The authors of outstanding presentations were encouraged to submit a manuscript to be published in this thematic series of the Journal of Neuroengineering and Rehabilitation (JNER). As a result, this thematic series includes six manuscripts highlighting major areas of ongoing research in the field of NR.

Three articles focused on the development of assessment strategies of relevance to the field of NR. Coscia et al. explored how changes in the amount of arm weight support affect muscle activation, kinematics and motor control during arm reaching movements in healthy volunteers. They analyzed the hand, elbow, shoulder and trunk trajectories, and the activation of fourteen muscles of the upper extremity to derive muscle synergies during arm reaching movements in six different conditions or arm weight support. They found that the same eight muscle synergies were consistently observed during the performance of arm reaching movements across different levels of arm weight support. The magnitude of the activation of individual muscles showed a decrease with an increase in the amount of arm weight support. The authors found variability in the kinematic of arm reaching movements across different levels of arm weight support, although no specific trend was associated with the amount of arm weight support.

Bravo-Esteban et al. investigated the potential of the electromyographic (EMG) coherence analysis of the tibialis anterior (TA) muscle as a measure of strength, quality of movement during gait and severity of spasticity in subjects with an incomplete spinal cord injury (iSCI). They explored the coherence value within specific frequency bands during isometric, isokinetic and isotonic controlled movements of the ankle. They found that intramuscular 15–30 Hz TA coherence value during isometric contractions generating maximal voluntary torque (MVT) was correlated to residual strength and gait function after iSCI, and that several spastic symptoms were negatively correlated with 10–16 Hz and 40-60Hz TA coherence value during isometric contractions at MVT.

Meyer et al. presented results suggesting that pre-trial brain electroencephalographic (EEG) data analysis can be used to predict performance in a reaching task in healthy subjects. In order to measure the learning process, the authors used the normalized time-to-target parameter, defined as the time required to reach the target from the verbal command to begin the movement, divided by the distance from starting to target position. The analysis of EEG data revealed the involvement of brain areas that also play a role in the motor learning process, and that the α/μ frequency band contains the most relevant information to predict intervention outcomes.

The remaining three articles are focused on the assessment of different treatment strategies in NR. Marchal-Crespo et al. assessed different robotic training strategies to evaluate the impact of movement error amplification and reduction on motor learning and muscle activation during the performance of a simple task. This study was performed with the robotic system MARCOS, suitable to be used simultaneously with Magnetic Resonance Imaging (MRI). Healthy subjects were asked to train under four control strategies: haptic guidance, no guidance, error amplification and noise disturbance while measuring muscle activation in addition to neuroimaging data. The authors found that strategies adding disturbances and amplifying error enhanced muscle activation and boosted motor learning.

Fleerkotte et al. evaluated the effects of an eight-week training program with a prototype of a robotic gait trainer (LOPES) in patients with iSCI, using an impedance control strategy. Improvements in functional outcomes were observed after training, which were retained at an eight-week follow-up. Significant improvements were also observed in the hip joint kinematics. The authors reported that participants showing pre-training lower walking function showed the largest relative improvements.

Finally, del-Ama et al. presented a cooperative control strategy for an hybrid exoskeleton (Kinesis) that leveraged robotic actuation and functional electrical stimulation of lower limb muscles. This control approach was tested in healthy subjects showing that the system is able to monitor muscle performance during gait to estimate and manage muscle fatigue while training with the Kinesis system. The authors argue that the system has potential for gait rehabilitation in patients with SCI.

Altogether, this thematic series present new approaches to evaluate CNS motor control and innovative treatment strategies in NR. Future research in this field should focus on providing evidence of usability and efficacy in the clinical settings, including a detailed description of the ideal conditions for application of these new NR technologies.
